# Reserve antibiotics: overcoming limitations of evidence generated from regulatory approval trials

**DOI:** 10.1186/s12992-025-01109-4

**Published:** 2025-04-03

**Authors:** Lorenzo Moja, Mohamed Abbas, Marlieke EA de Kraker, Veronica Zanichelli, Loice Achieng Ombajo, Mike Sharland, Benedikt Huttner

**Affiliations:** 1 Health Products Policy and Standards, World Health Organization, Geneva, Switzerland; 2 Infection Control Programme, Geneva University Hospitals and Faculty of Medicine, Geneva, Switzerland; 3 WHO Collaborating Centre on Patient Safety, Geneva, Switzerland; 4 Department of Clinical Medicine and Therapeutics, School of Medicine, University of Nairobi, Nairobi, Kenya; 5 Centre for Neonatal and Paediatric Infections, Institute for Infection and Immunity, St George’s University of London, London, UK; 6 Division of Antimicrobial Resistance, World Health Organization, Geneva, Switzerland

**Keywords:** Multiple bacterial drug resistance, Comparative effectiveness research, Anti-bacterial agents, Clinical trials, Antimicrobial stewardship

## Abstract

New antibiotics active against multidrug resistant bacteria (MDR-B) are licensed by regulatory agencies based on pivotal trials that serve the primary purpose of obtaining marketing-authorization. There is increasing concern that they do not offer guidance on how to best use new antibiotics, in which population, and to what extent they overcome existing resistance. We reviewed the literature for pre-approval studies (phase 2 and 3 randomized controlled trials) and post-approval studies (randomized and non-randomized controlled trials) evaluating efficacy and safety of new antibiotics, classified by WHO as Reserve, approved in the European Union and the US from January 2010 to May 2023. Substantial failures occur in generating evidence to guide routine clinical use: preapproval studies lack representativeness, select outcomes and comparators to chase statistical significance, and often avoid using prespecified analytical methods. Three recommendations are key to enhance the quality and relevance of clinical data underpinning use of last resort molecules on the WHO AWaRe Reserve list active against carbapenem-resistant MDR-B i). separation of pivotal trials from post-approval studies, which should be funded by public programs and de-linked from commercial purposes, ii). development and maintenance of a global infrastructure to conduct post-approval public health focused studies, and iii). development of trial platforms that use efficient, adaptive designs to inform clinical decision making and country level technology appraisal. These solutions will allow clinicians to determine whether recently approved Reserve antibiotics are not only “newer” but also “better” for vulnerable patient populations at particular risk for infections by MDR-B.

## Introduction

Antibiotic resistance has been estimated to be directly responsible for 1.27 million deaths globally in 2019 [1]. The balance between the need to both develop and rapidly approve novel antibiotics to increase the therapeutic armamentarium against potentially life-threatening infections caused by multidrug resistant bacteria (MDR-B) and the need to generate evidence regarding their efficacy, safety and appropriate clinical use is often skewed toward the former [2, 3]. The generation of relevant evidence for novel antibiotics active against MDR-B is further complicated by limited commercial prospects compared to other medicines [4]. Against this scenario national and international agencies have invested heavily to explore novel incentive models and international coordination to reinvigorate the antibacterial R&D [5]. However, these financial initiatives will have limited long-term public health benefit if the clinical studies performed do not inform their routine optimal use.

In 2017 the World Health Organization (WHO) introduced a new classification, categorizing antibiotics into three groups: **A**ccess, **Wa**tch, and **Re**serve (AWaRe) [6, 7]. Reserve antibiotics were defined as “last resort antibiotics” [8], the use of which should generally be limited to targeted treatment of specific indications and patients, in the absence of alternatives. Among the 29 antibiotics currently classified by WHO as “Reserve” 12 have been approved by FDA and EMA since 2010 and most of these (*n* = 8/12) have in vitro activity against carbapenem-resistant “critical priority” MDR-B in the WHO Priority Pathogens List [9]. Reserve antibiotics listed on WHO’s Model Lists of Essential Medicines (EML) should be accessible to patients who need them, while also being a key target for antibiotic stewardship programs to prevent emergence and spread of resistance to these and other antibiotics. This poses considerable challenges in many settings, notably where the microbiologic diagnostic infrastructure is unavailable to reliably identify patients with MDR-B infections or colonization.

Use of Reserve antibiotics should be in accordance with international recommendations for the treatment of MDR-B [10, 11]. WHO has recently issued guidance for the optimal use of the Reserve antibiotics on the EML [12]. However, the development of evidence-based guidance for the appropriate clinical use of these antibiotics for the treatment of suspected or confirmed MDR-B infections is hampered by the lack of high-quality evidence. A classic scenario encountered in this context is that of approval of a new Reserve antibiotic based on a phase 3 trial studying the novel antibiotic in patients with complicated urinary tract infections, without a specific focus on infections caused by MDR-B. This is then occasionally followed by a single, small, often non-randomized post-approval study testing the new agent specifically in patients with infections caused by MDR-B [13–17]. While attempts to increase the incentives for the development of new antibiotics are critically important, they also need to ensure that the evidence generation for these antibiotics meets patients’ and public health needs [17]. In this article we examine the current status of evidence generation for new Reserve antibiotics at two successive research stages - pre and post approval - and consider how the evidence generation can be improved to align with patients’ and public health needs.

## Methods

Our analysis was informed by a detailed breakdown of the problems with pre-approval randomized controlled trials (RCTs) and post-approval randomized and non-randomized trials that assessed efficacy and safety of Reserve antibiotics approved in the European Union and the US between January 2010 and May 2023, namely cefiderocol, ceftazidime-avibactam, ceftolozane-tazobactam, eravacycline, imipenem-relebactam, meropenem-vaborbactam, and plazomicin. In these trials comparators were usually non-Reserve antibiotics, such as carbapenems. Through discussions among members of the study team and by tapping its collective experience, we identified areas of improvements and suggestions that are likely to improve the evidence generation on Reserve antibiotics across a range of acute infections.

## Results

**Fig. 1:**
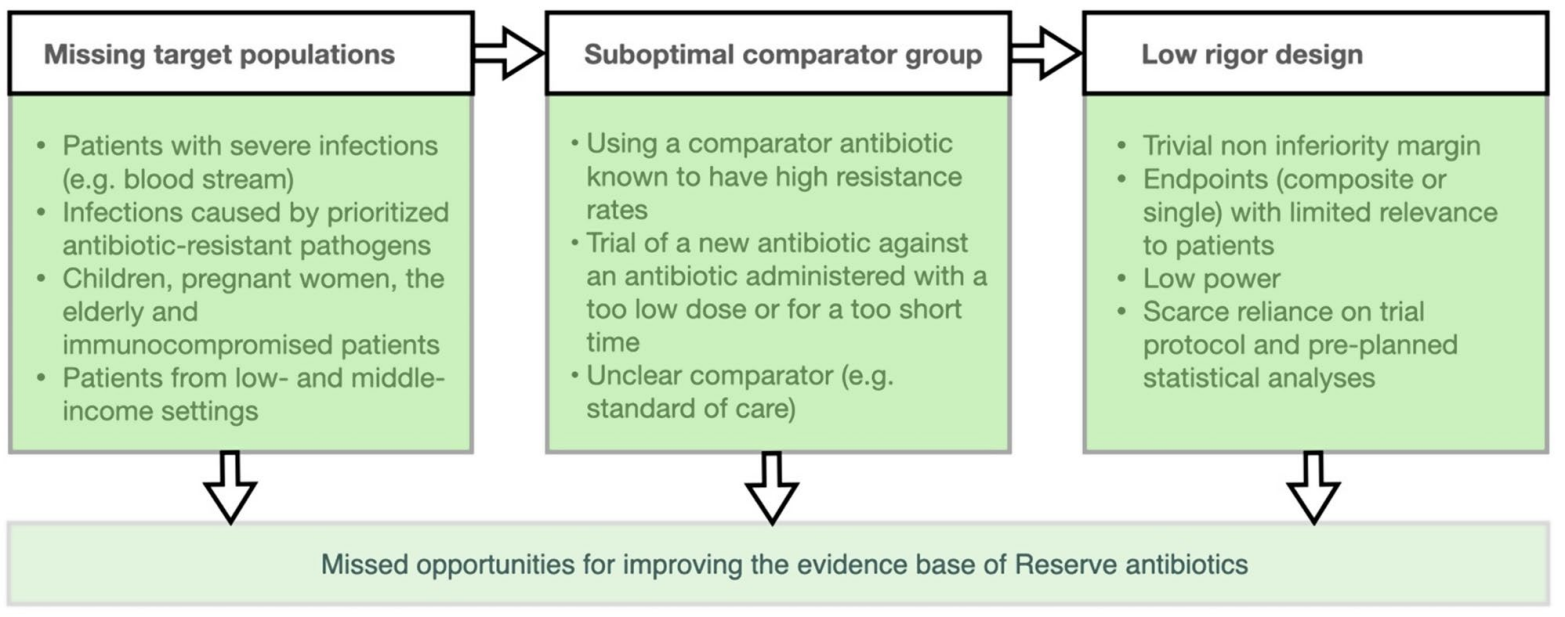
Main challenges of trials testing Reserve antibiotics that reduce generalizability of results to real-world practice

We identified 10 phase 2 RCTs, 19 phase 3 RCTs, and 5 post-approval randomized and non-randomized trials that assessed the efficacy and safety of the Reserve antibiotics mentioned above (Table 1 summarises key features across phase 3 RCTs while Table 2 provides details of each phase 3 RCT). While the number of phase 3 RCTs per antibiotic was limited (median 2, range 2–5), when all trials were considered together, several characteristics and patterns in their design emerged, offering a clear contrast between the actual research output and the desirable high-quality, actionable evidence that is needed to guide their clinical use. We identified six areas for improvement regarding both pre-approval randomized clinical trials and post-approval studies (Fig. 1).

**Table 1:** Summary description across included phase 3 RCTs evaluating efficacy and safety of new Reserve antibiotics

Characteristic	Category	Phase 3 RCTs (*N* = 19) *n* (%)
Antibiotic investigated	Cefiderocol	2 (10.5)
	Ceftazidime-avibactam	5 (26.3)
	Ceftolozane-tazobactam	4 (21.1)
	Eravacycline	2 (10.5)
	Imipenem-relebactam	2 (10.5)
	Meropenem-vaborbactam	2 (10.5)
	Plazomicin	2 (10.5)
Clinical syndrome*	cUTI and/or pyelonephritis	8 (42.1)
	cIAI	9 (47.4)
	HABP and/or VAP	8 (42.1)
	BSI	3 (15.8)
Type of study	Non-inferiority trial	14 (73.7)
	NI margin 10%	6 (31.6)**
	NI margin 12.5%	7 (36.8)**
	NI margin 15%	2 (14.3)**
	Descriptive study without inferential/hypothesis testing	5 (26.3)
WHO regions*	African Region †	5 (26.3)
	Region of the Americas	17 (89.5)
	Eastern Mediterranean Region	1 (5.3)
	European Region	17 (89.5)
	South-East Asian Region	6 (31.6)
	Western Pacific Region	13 (68.4)
Inclusion of special populations	Infections due to CR-GNB (> 50% patients)	4 (21.1)
	Pregnant females	0 (0)
	Paediatric patients	0 (0)

BSI, bloodstream infection; cIAI, complicated intra-abdominal infection; CR-GNB, carbapenem-resistant gram-negative bacteria; cUTI, complicated urinary tract infection; HABP, hospital-acquired bacterial pneumonia; VAP, ventilator-associated pneumonia

* Totals exceed 100% because some studies investigated > 1 clinical syndrome

** Totals exceed 100% because some studies had 2 co-primary endpoints with > 1 non-inferiority margin

† Only South Africa

**Table 2:** Characteristics of the included phase 3 RCTs evaluating efficacy and safety of new Reserve antibioticse

Author Year Trial acronym	Antibiotic Comparator	Design	Infection(s)	Total number of participants (% of patients with antimicrobial resistance GNB by microbiological diagnosis)	Primary outcome
Bassetti 2021 CREDIBLE-CR [20]	cefiderocol BAT	exploratory analysis with no hypothesis testing	HAP, VAP, HCAP, BSI or sepsis, cUTI	152 (98.7% CR-GNB)	clinical cure
Carmeli 2016 REPRISE [78]	ceftazidime-avibactam BAT	exploratory analysis with no hypothesis testing	cUTI, cIAI	333 (76.5% ceftazidime resistant GNB)	clinical cure
Kaye 2018 TANGO II [41]	meropenem-vaborbactam piperacillin-tazobactam	non-inferiority (with 15% non-inferiority margin)	cUTI	550 (0.5% CR-GBN; 6.7% piperacillin-tazobactam)	FDA: composite of clinical cure and microbiologic eradication; EMA: microbiological eradication
Kollef 2019 ASPECT-NP [79]	ceftolozane-tazobactam meropenem	non-inferiority (with 10.0% non-inferiority margin, one sided significance level)	HAP, VAP	726 (*P. aeruginosa* MDR and XDR 6.8% and 2.9%; meropenem resistant 13%; ceftolozane-tazobactam resistant 3%; Enterobacteriaceae meropenem resistant 1%; ceftolozane-tazobactam resistant 13%)	composite of clinical cure and microbiologic eradication
Mazuski 2016 RECLAIM 1 and 2 [80]	ceftazidime-avibactam plus metronidazole meropenem	non-inferiority (with 12.5% non-inferiority margin)	cIAI	1066 (13.5% ceftazidime resistant GNB)	clinical cure
McKinnell 2019 CARE [21]	plazomicin colistin	exploratory analysis with no hypothesis testing, early stopped for slow enrolment	BSI, HAP/VAP	39 (94.8 CR-GNB)	composite outcome of all-cause mortality or clinically significant disease-related complications
Motsch 2020 RESTORE-IMI 1 [22]	imipenem-relebactam colistin-imipenem	exploratory analysis with no hypothesis testing	HAP, VAP, cUTI, cIAI	47 (66.0 CR-GNB)	favourable overall response
Qin 2017 RECLAIM 3 [81]	ceftazidime-avibactam plus metronidazole meropenem	non-inferiority (with 12.5% non-inferiority margin)	cIAI requiring surgical intervention	486 (18.6% ceftazidime resistant GNB)	clinical cure
Solomkin 2015 ASPECT-cIAI [82]	ceftolozane-tazobactam plus metronidazole meropenem	non-inferiority (with 10.0% non-inferiority margin)	cIAI	993 (7.2% ESBL-producing GBN)	clinical cure
Solomkin 2017 IGNITE 1 [83]	eravacycline ertapenem	non-inferiority (with 10.0% non-inferiority margin)	cIAI requiring surgical intervention	541 (2.9% CR-GNB)	clinical cure
Solomkin 2019 IGNITE 4 [84]	eravacycline meropenem	non-inferiority (with 10.0% non-inferiority margin, one sided significance level)	cIAI	500 (6.8% CR-GNB)	clinical cure
Sun 2022 MK-7625 A [85]	ceftolozane-tazobactam (plus metronidazole) meropenem (plus placebo)	non-inferiority (with 12.5% non-inferiority margin, one sided significance level))	cIAI	268 (11.9% ESBL positive [18.2% of isolates])	clinical cure
Titov 2020 RESTORE-IMI 2 [86]	imipenem-relebactam piperacillin/tazobactam	non-inferiority (with 12.5% non-inferiority margin, one sided significance level))	HAP, VAP	537 (not reported)	all-cause mortality
Torres 2018 REPROVE [87]	ceftazidime-avibactam meropenem	non-inferiority (with 12.5% non-inferiority margin)	HAP, VAP	879 (28.2% ceftazidime resistant GNB)	clinical cure
Wagenlehner 2015 ASPECT-cUTI 1 and 2 [40]	ceftolozane-tazobactam levofloxacin	non-inferiority (with 10.0% non-inferiority margin)	cUTI	1083 (2.7% ceftolozane-tazobactam resistant GNB; 26.5% levofloxacin resistant GNB)	clinical cure
Wagenlehner 2016 RECAPTURE 1 and 2 [48]	ceftazidime-avibactam doripenem	non-inferiority (with 10% non-inferiority margin for FDA and 12.5% for EMA)	cUTI	1033 (19.6% ceftazidime resistant GNB)	FDA: symptomatic resolution; both microbiological eradication and symptomatic resolution. EMA: microbiological eradication
Wagenlehner 2019 EPIC [88]	plazomicin meropenem	non-inferiority (with 15% non-inferiority margin)	cUTI	609 (2.5% CR-GNB; 18.8% multidrug resistant)	clinical cure and microbiologic eradication
Wunderink 2018 TANGO II [23]	meropenem-vaborbactam BAT	exploratory analysis with no hypothesis testing	cUTI/AP, HAP, VAP, BSI, cIAI	77 (70.1% CR-GNB)	composite of clinical cure and microbiologic eradication, all-cause mortality
Wunderink 2021 APEKS-NP [43]	cefiderocol meropenem	non-inferiority (with 12.5% non-inferiority margin)	HCAP, HAP, VAP	300 (15% CR-GNB)	all cause-mortality

BAT: best available therapy; BSI: bloodstream infections; cIAI: complicated intra-abdominal infection; CR-GNB: carbapenem-resistant gram-negative bacteria; cUTI: complicated urinary tract infection; ESBL: extended-spectrum beta-lactamase; HAP: hospital-acquired pneumonia; HCAP: healthcare-associated pneumonia; VAP, ventilator-associated bacterial pneumonia

### Challenge 1: representativeness

By definition Reserve antibiotics should be “reserved” for patients with infections caused by MDR-B (targeted use) or for patients with a high probability of a severe infection by MDR-B (empiric use) [18, 19]. However, 14 of the 19 identified phase 3 trials did not specifically include patients with documented or suspected infections caused by these highly resistant pathogens, therefore there is limited evidence about clinical efficacy against MDR-B infections. Most studies examined empiric treatment of infections (i.e. before susceptibility results for the causative organism were available) in patient populations unselected for their risk of carbapenem-resistant gram-negative bacteria (CR-GNB) infections. Only a minority (4/19, 21%) used documentation of an infection by CR-GNB as an inclusion criterion [20–23]. In phase 3 trials, the proportion of patients with documented infections due to carbapenem-resistant Gram-negative bacteria (CR-GNB) ranged from 1.4% (eravacycline) to 36.5% (cefiderocol) (Tables 1 and 2).

The identification of a microbiologically confirmed resistant bacterial phenotype is often not used as a trial inclusion criterion. Patients are indeed included based on clinical severity (e.g. age, renal function) more than risk for AMR. This easement of eligibility requirements facilitates recruitment but dilutes the clinical relevance of efficacy results. It leads to the inclusion of patients with infections with less difficult-to-treat phenotypes of resistance for which antibiotics in lower levels of the WHO’s AWaRe classification (i.e. Access and Watch) would often be effective with no need for last-resort Reserve antibiotics. The other problem is that these results do not reduce uncertainty on how to identify patients that would benefit most from new antibiotics (e.g. patients with infections caused by carbapenem-resistant bacteria). In “real life”, given the limited number of alternatives, Reserve antibiotics may be mostly used for targeted treatment, when the target pathogen and its resistance profile are known.

Vulnerable patient populations most affected by infections due to MDR-B such as neonates, elderly patients, severely immunocompromised, or patients with multiple comorbidities were either not included or underrepresented. For instance, only 2 RCTs included paediatric populations (3 months to 18 years), both testing ceftazidime-avibactam, resulting in the FDA’s approval for use in children > 3 months for complicated urinary tract infection and complicated intra-abdominal infection [24, 25]. Unfortunately, the burden of colonization and severe infections caused by MDR-B is high in neonates, particularly in low- and middle-income settings, and associated with increased mortality [26–30]. The lack of evidence supporting antibiotic use in neonates and small children has led to 50% of antibiotics (not limited to Reserve antibiotics) being prescribed off-label in European countries in this population [31]. In addition, no single trial has evaluated the safety for both mother and child of these new antibiotics in pregnant and/or lactating female patients although there are no clear reasons against the inclusion of this population in trials testing antibiotics. All mentioned flaws limit the representativeness of trial results for those high-risk patients that would mostly benefit from new treatments [32–35]. 

### Challenge 2: choice of primary outcomes

**Fig. 2:**
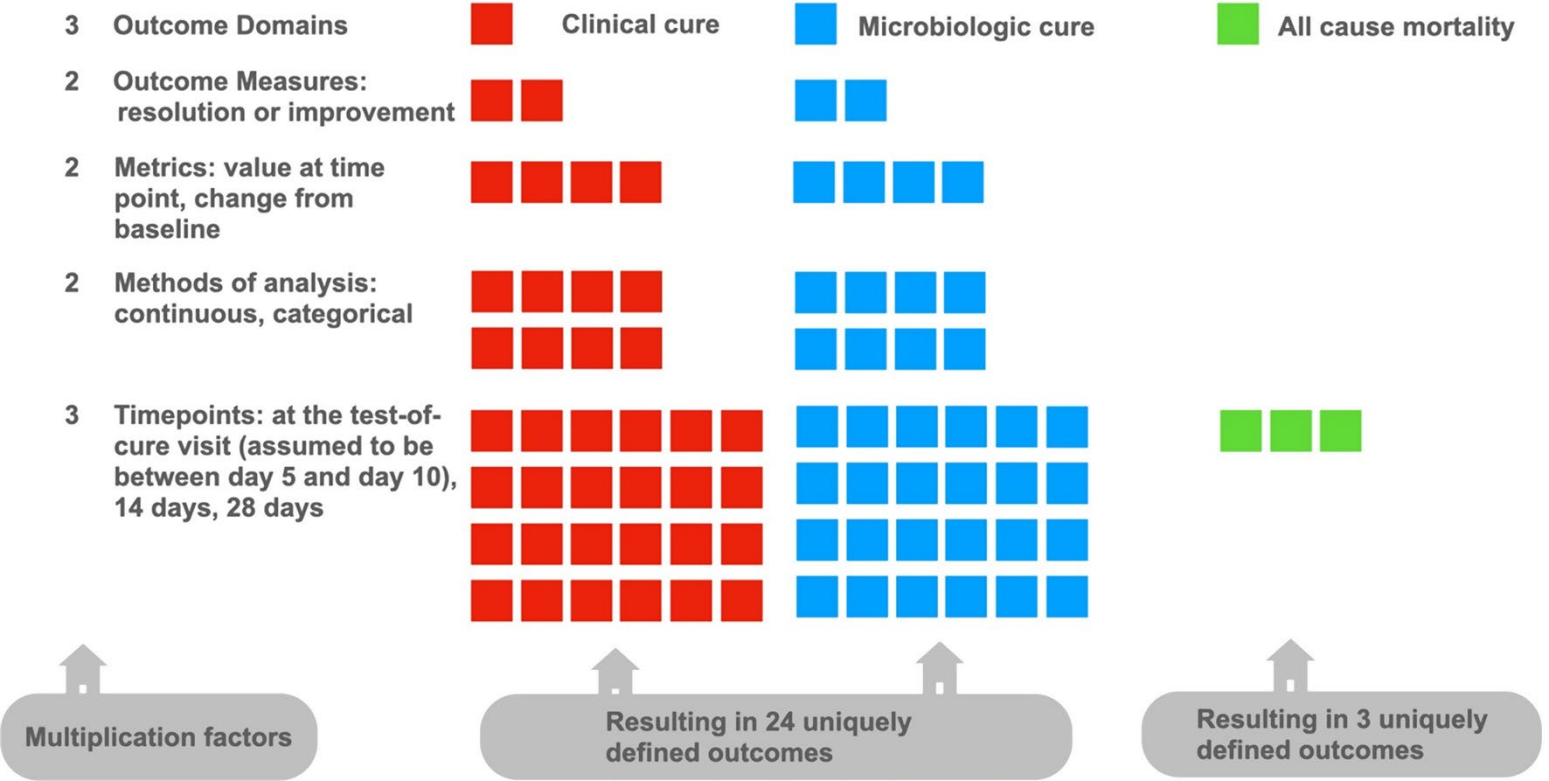
Overview of variations in elements of primary outcomes used in pivotal trials for Reserve antibiotics and the resulting number of possible, differently defined primary outcomes based on combinations of these elements. Footnote: The number of outcomes in trials testing Reserve antibiotics is a function of the number of definitions of each of multiple element that characterizes the outcome. In this figure we report the number of outcomes that is hypothetically usable if all combinations of definitions of the outcomes proposed in in the cohort of included RCTs are used. This estimate is conservative as it does not include composite endpoints. Squares represent elements that influence the final number of outcomes. Colours are decorative

The primary outcomes of the RCTs are often of uncertain reliability [36] even when they are prespecified as part of the engagement between pharmaceutical companies and regulatory authorities. In all selected trials, primary outcomes could be aggregated into three main domains: clinical cure, microbiological cure, and mortality (Fig. 2). For each outcome domain under clinical and microbiologic cure, RCTs may include many different outcomes because of the different measures, metrics, methods of aggregation, and time points used. An outcome can be defined according to the following elements:

- domain: e.g., microbiologic cure;
- specific measure: e.g., pathogen reduced to < 10^4^ colony forming units (CFU/ml) in urine;
- metrics: e.g., change from baseline;
- methods of analysis: e.g., microbiologic intention-to-treat population;
- timepoint: e.g., test-of-cure visit.

Each element multiplies the number of potential primary outcomes. In studies of complicated urinary tract infections, abdominal infections, and bloodstream infections, the primary outcomes were markedly heterogeneous in the definition of microbiologic and clinical cure outcomes or both combined in one outcome. Heterogeneity in primary outcomes selection reduces the possibility of aggregating evidence across multiple studies in meta-analyses [37]. In addition, surrogate outcomes such as “microbiologic response” are often imperfect predictors of clinical outcomes. Where clinical outcomes were used, shortcomings such as a high degree of subjectivity (e.g. significant improvement of baseline signs) were frequent, which is a special concern if the study is unblinded [38]. Several trials used composite endpoints (e.g. clinical cure plus microbiologic eradication) which can often lead to exaggerated estimates of antibiotic efficacy, particularly when microbiologic outcomes are used as part of the outcome combination [39]. 

While introducing a certain degree of flexibility in defining outcomes can be desirable, this flexibility can have unintended consequences if it conceals the clinical relevance of findings, limits the comparison of trial outcomes and ultimately misleads users. Short-term mortality is an objective outcome that can be interpreted and compared between studies with high confidence [36]. Despite its prominent role this outcome is ignored in most pivotal trials. Therefore, the reliability of primary outcomes of RCTs is often uncertain, even when they are prespecified as part of the engagement between pharmaceutical companies and regulatory authorities [36]. 

### Challenge 3: choice of comparator(s)

The choice of comparator is crucial for RCTs evaluating novel treatments and can heavily influence its conclusions. If a suboptimal comparator treatment is chosen, it becomes both more likely for a medicine to demonstrate “non-inferiority” or “superiority”. In a RCT comparing ceftolozane-tazobactam to levofloxacin for complicated urinary-tract infections (cUTI), the prevalence of resistance to levofloxacin in the urinary pathogens was– unsurprisingly - tenfold higher than to ceftolozane-tazobactam [40]. Demonstrating superiority with regard to microbiological clearance, the EMA primary outcome, thus became a kind of “self-fulfilling prophecy”. This also raises questions regarding the study design: in settings with a high prevalence of fluoroquinolone resistance levofloxacin would not have been considered an acceptable empiric treatment option. Differences in dosing between the study arms can also favour the new study drug. In the study comparing meropenem-vaborbactam to piperacillin-tazobactam for cUTI, the study antibiotic was administered in extended perfusion over 3 h, whereas the comparator was administered in short perfusion putting the comparator potentially at a disadvantage since both are time-dependent antibiotics [41, 42]. Trial protocols aiming at optimising the pharmacokinetic and pharmacodynamic properties of the comparator antibiotic are possible: e.g. in the phase 3 trial comparing cefiderocol to meropenem, the latter was administered at high doses and with extended-infusion protocols [43]. In general, dosing strategies for the comparator should be optimized (be it the total dose administered, the frequency of dosing or the time of infusion) as demonstrating non-inferiority or even superiority relative to an ineffective or substandard control severely limits the usefulness of the generated data. Said this, it is possible that in LMIC settings, the selection of the best target antibiotic is a factor of limited importance when compared to delayed access to health care and absence of life-support therapies.

### Challenge 4: Pre-planned Inferential testing of the main hypothesis

All described trials aimed to compare efficacy of different antibiotics but often they lacked the operational definition of the procedure to test this difference. Pre-specification of the planned statistical analysis approach is essential to help reduce bias associated with investigators selecting their analysis method after seeing the trial results and “cherry picking” favourable analyses. Several studies (Table 2) did not provide details of the methods of analysis of primary outcomes. For instance, none of the RCTs focused on CR-GNB infections reported a pre-planned superiority analysis or presented pre-planned inferential testing [20–23]. 

One of these studies compared meropenem-vaborbactam to a heterogeneous comparator - “best available therapy” - in patients with confirmed infections by carbapenem-resistant Enterobacterales (mostly in the context of urinary tract infections and bacteraemia) and demonstrated superiority of the new antibiotic [23]. When facing MDR-B it may be difficult to privilege only one comparator as it is difficult to choose the best treatment. As a result, the trial control arm often is highly heterogenous in terms of antibiotics used, limiting our interpretation of the final results. Mitra-Majumdar et al. point out that, irrespective of calculations of power needed to accept the null hypothesis conventionally established by FDA, the results of one third of trials testing new antibiotics are uninterpretable [44]. To increase the interpretability of trial results in relation to the control arm, an alternative tactic is to assess patients’ eligibility based on very few pre-specified therapeutic options for the control arm. If the treatments in the control arm are not appropriate for that patient, trialists can opt to not randomise the patient.

Other aspects of the analyses are often not documented, such as prevention and handling of missing data. Excluding patients with missing data or using suboptimal imputation methods may bias the results [45]. When statistically significant results are obtained in a “methodological vacuum” the confidence in the accuracy of the trial results is decreased.

### Challenge 5: Non-inferiority statistical hypotheses with often large non-inferiority margins

Most studies adopt a non-inferiority design aimed at demonstrating that the study drug is not worse than the comparator (Tables 1 and 2). The wide use of noninferiority design is due to the interplay of two causes. In a setting where a new antibiotic needs to be compared to standard-of-care antibiotics with a high efficacy for infections caused by susceptible bacteria, in a patient group with mostly drug-susceptible infections, demonstration of statistical superiority becomes unlikely [46]. Regulatory agencies prefer trials in which prior antibiotic therapy is not allowed [47], while selection of patients with infections caused by MDR-B requires antimicrobial susceptibility results that mostly take > 48 h after the sample is taken, during which time empirical treatment is often already started. Therefore, the number of eligible patients with infections caused by MDR-B is often limited, leading to large non-inferiority margins.

Non-inferiority margins are set by regulatory agencies, and currently range from an absolute difference in efficacy of no more than 10 to 20% points for pivotal antibiotic treatment trials, depending on the infection, with a preference for 10% [47]. This margin might expose patients to clinical experiments that could result into registration of novel antibiotics that do not improve patient care, or even result in a worse outcome. Moreover, the risk with non-inferiority hypotheses is that poor or flexible conduct of the trial, or non-adherence to the experimental antibiotic, can falsely increase the chance of claiming non-inferiority.

In theory, as current standard-of-care treatments for infections caused by MDR-B have only suboptimal efficacy, demonstrating superiority should be feasible. Three studies adopting a non-inferiority design demonstrated that the new antibiotic was superior to the comparator in at least one of the primary endpoints. For instance, in a study comparing ceftazidime-avibactam to doripenem in complicated urinary tract infection, ceftazidime-avibactam demonstrated superiority in one of the co-primary outcomes, which was microbiological eradication at the test-of-cure visit (EMA primary endpoint), but not in the other co-primary endpoints (FDA co-primary endpoints of patient-reported symptomatic resolution at day 5 and combined symptomatic resolution/microbiological eradication at test-of-cure visit) [48]. It is likely that investigators are now privileging a risk mitigation tactic preferring to demonstrate non-inferiority of a new antibiotic rather than aiming at establishing its superiority. Demonstrating non-inferiority will require a smaller difference in treatment effect but this may still be sufficient to support market product approval. However, Outterson et al. pointed out that 43% (26/61) of antibiotics approved between 1980 and 2009 were withdrawn by 2013, often based on safety concerns [49]. From 1980 to 2019, the large increase of antibiotics approved on the basis of non-inferiority trials was accompanied by a drop in the number of antibiotics indicated for serious and life-threatening diseases [50], with at least two trials showing an increase in mortality with the new antibiotics [20, 22]. Where superiority trials are not feasible, greater care should be taken to assure that inferences about non-inferiority are valid and acceptable for patients and prescribers.

### Challenge 6: very limited information from post–approval studies

There were few comparative post-approval studies, all nonrandomised, concerning only 2 of the antibiotics: ceftazidime-avibactam (*n* = 3) [51–53] and ceftolozane-tazobactam (*n* = 2) [54, 55]. Among the 5 post-approval observational studies, 3 were not funded by pharmaceutical companies keeping the testing at arm’s-length from commercial interest, one being funded by the Italian Ministry for University and Scientific Research [52] and one by the US National Institute of Allergy And Infectious Diseases [53] (the exact funding source of the third study was unclear). However, the role of follow-on studies in completing evidence generated by pivotal trials is not clear. Their limitations have been recently discussed, highlighting how they rarely solve questions and doubts generated at the time of approval in relation to safety concerns, potential to favour the selection of resistant pathogens, or efficacy in special populations, including resistant infections [44]. Furthermore, all nonrandomized studies were at risk of confounding by indication, limiting any inference on efficacy. As happened in the observational studies conducted during the covid-19 pandemic, the problem is that most findings of observational studies could not be replicated in large RCTs [56]. Reflecting on the anticipated information value of a post-approval study should guide decisions about whether it is reasonable to initiate or fund a particular study in terms of reducing uncertainty around antibiotic efficacy. Removing randomization will not help to generate credible evidence but might help disseminating treatment that turns out to be ineffective or deleterious.

### Options for improvement: separating pivotal trials from post-approval evidence generation studies

As a result of the limitations of the most recent generation of trials of new antibiotics, there has been a push by public health advocates towards generating more high-quality actionable evidence for the use of these new medicines [57]. We have identified the post-approval space where public health evidence generation should be focused.

The pre-approval components involve acting within the negotiation that takes place between regulatory agencies and industry, a step in which there is often limited scope for intervention by the scientific community. Paul et al. recommended that industry-led trials should be avoided altogether, in favour of clinical trial networks run by academic investigators [58]. The authors argue that the high cost of running industry-sponsored trials is not justified by the apparent advantages over investigator-initiated trials. In fact, it is proposed that there is a lower risk of bias and improved external validity of investigator-initiated trials. We agree that antibiotic research can benefit from a distinct separation of two translational complementary blocks [59]: the first allows for timely approval of promising new antibiotics that can be commercialized (“brought to market”); the second translates research into practice; i.e. ensuring that new antibiotics actually address the patients or populations for whom they are intended and that their use is implemented correctly. The second block of translational research seeks to minimize some of the discussed biases, reorganizing the evidence base and synergies of the health care decision-making ecosystem [60]. This separation would limit potential concerns from drug companies: setting a higher bar for the approval of novel antibiotics for the few pharmaceutical companies still active in the development of antibiotics may be counterproductive without concomitant novel incentive mechanisms [61, 62]. Antibiotics are less profitable for pharmaceutical companies than other products, thus representing an “opportunity cost” in terms of time and resources [63], especially since uncontrolled use of Reserve Group antibiotics is strongly discouraged internationally in an effort to curb antibiotic resistance.

An example of a post-approval trial to increase knowledge about the use of an essential Reserve antibiotic, cefiderocol, is the “GAME CHANGER”. This non-inferiority trial assessed short-term all-cause mortality in adult patients with healthcare-associated and hospital-acquired GNB infections treated with cefiderocol or standard of care (e.g., colistin). In order to include 120 patients with carbapenem-resistant GNB infections (out of approximately 500 patients included overall), over 9000 patients were screened for eligibility. Cefiderocol was shown to be non-inferior and not superior to standard of care. Mortality in the subgroup of carbapenem-resistant infections was higher in the cefiderocol arm (although not statistically significant), raising some concern particularly since a number of patients in the control group received treatment with carbapenems [64]. The GAME CHANGER trial design provides helpful experience in the challenges of designing public health trials assessing the “real-life” added clinical value of these new agents.

### Options for improvement: funding the development and maintenance of post-approval research infrastructure

From a system perspective, there must be public contribution to the efforts of generating better evidence for future appraisals of new antibiotics than the current registration trials [65]. Pull incentives to trigger investment in the development of new Reserve antibiotics, such as the recent PASTEUR Act and AMR Action Fund [66], should be complemented with funding to conduct non-industry-sponsored, larger, more complex, trials that ensure the generation of real-life data on the added clinical value of these new treatments. An important element of post-approval research is that the focus of the study is the clinical infection, rather than a single investigational drug (as in a classic two-arm RCT), where multiple investigational and comparator drugs can be assessed. These trials are often too complex to be planned and conducted by single researchers or small academic centres. Multiple challenges can be encountered which investigators cannot fully control or rapidly solve from lack of research infrastructure and resource constraints to organizational inertia that paralyzes an organization from making changes needed for a more effective conduct of trials. However, this strategy has been successfully implemented for COVID-19 [67]. For instance RECOVERY compared multiple treatment arms with a shared single control group, using factorial randomisation, in which patients are randomised to active treatment or usual care independently for each of the suitable interventions [68]. Recent initiatives such as the European Clinical Research Alliance on Infectious Diseases (ECRAID) project are welcomed attempts to generate such infrastructure, aggregating academic centres that have the necessary expertise to successfully design, implement and analyse new antibiotic trials [69]. 

### Options for improvement: international trial platforms at scale

There is a clear need to develop a platform trial infrastructure, which has three key characteristics: (1) adaptive RCTs in which multiple antibiotics are evaluated against clinical syndromes in a perpetual manner, (2) antibiotics allowed to enter or leave the platform on the basis of a strategic algorithm, and (3) being outside regulatory approval pathways. The research community is still defining the best strategy to be adopted by trial platforms. An independent group of authors have advocated for the use of superiority adaptive RCTs with superiority demonstrated by reference to a one-sided significance test (as usually the investigational drug is already established as non-inferior), and a “three-stage sequential design” with 2 interim analyses and a final analysis [14]. The analyses at each stage allow for early-stopping based on prespecified rules in case efficacy is already attained, as well as reassessment of the sample size, should the assumptions in the initial calculation not be upheld. This study design may decrease sample size requirements by 40% compared to a standard RCT. The STAT-Net group, part of the Combatting Bacterial Resistance in Europe (COMBACTE) consortium, recommended the use of alternative outcome measures, specifically, rank-based composite end points, which include both patient-centred outcomes (e.g. mortality) and syndrome-specific outcomes (e.g. clinical cure or disease-free days) to reduce the required sample size and make superiority trials more feasible [70]. One of the phase 3 RCTs reviewed (the only one to do so) evaluated the efficacy of ceftazidime-avibactam using desirability of outcome ranking and response adjusted for duration of antibiotic risk [53, 71]. This approach has, however, encountered criticism because of its complexity and the uncertainty regarding some of the underlying assumptions [72]. A further concept is the Personalised RAndomised Controlled Trial (PRACTical) design which proposes to extend a network meta-analysis approach to individual randomisation of patients where each eligible patient is randomly assigned one of several clinically acceptable treatment regimens (based on microbiologic results, toxicity, physician judgement etc.) from a personalised randomisation list [37]. It is argued that this approach would lead to trials more closely resembling “real-life” practice by allowing the inclusion of broader patient populations. This design is now being used in a global RCT sponsored by the Global Antibiotic Research and Development Partnership (GARDP) comparing novel combinations of older generic antibiotics to existing WHO recommended and other antibiotic regimens to treat neonatal sepsis, NeoSep1 [73]. Point-of-care trials have been also proposed as a potential solution [74, 75]. However, point-of-care trials do not adopt any particular trial design, but rather the emphasis is on integrating clinical research into routine health care delivery.

## Conclusions

Developing new antibiotics is a long and expensive process with no guarantees of success: most new molecules will never make it past phase I trials. Moreover, antibiotic use leads to resistance which over time reduces their monetary value. Financial incentives to developers for delivering products with characteristics specified by the funder represent an important leverage to motivate the development of new antibiotics. Requirements to access these incentives should not be perceived as too soft and favourable to prevent rewarding molecules with limited clinical utility based on evidence derived from uninformative studies.

Most of the current evidence for the treatment of patients with severe or life-threatening infections caused by MDR-B is insufficient to define the optimal use of new Reserve antibiotics compared to already available alternatives. This is all the more concerning considering that these antibiotics are being included in treatment guidelines [10], that some of them (e.g. ceftazidime-avibactam) are already widely used [76], while they are considerably more expensive compared to existing generic antibiotics [77]. The problems we identified often apply to trials for other medicines (including non-Reserve antibiotics) and should be addressed to find the right balance between the need to simplify trials (design and regulation) while getting clinically meaningful results to support a change in practice. To prioritize expanded access programs to those antibiotics that potentially treat life-threatening infections and produce strong evidence to reduce the unnecessary use of new antibiotics we need the involvement of governments and their funding agencies to give impetus to develop a dedicated research infrastructure.

## Data Availability

No datasets were generated or analysed during the current study.

## Abbreviations

AWaRe: Access, Watch and Reserve
BAT: Best available therapy
BSI: Bloodstream infections
CFU: Colony forming units
cIAI: Complicated intra-abdominal infection
CR-GNB: Carbapenem-resistant Gram-negative bacteria
cUTI: Complicated urinary-tract infections
EMA: European Medicines Agency (EMA)
ESBL: Extended-spectrum beta-lactamase
FDA: Food and Drug Administration
HAP: Hospital-acquired pneumonia
HCAP: Healthcare-associated pneumonia
MDR-B: Multidrug resistant bacteria
RCT: Randomised clinical trial
VAP: Ventilator-associated bacterial pneumonia
WHO: World Health Organization
